# Factors associated with increased risk of progression to respiratory syncytial virus-associated pneumonia in young Kenyan children

**DOI:** 10.1111/j.1365-3156.2008.02092.x

**Published:** 2008-07

**Authors:** Emelda A Okiro, Mwanajuma Ngama, Ann Bett, Patricia A Cane, Graham F Medley, D James Nokes

**Affiliations:** 1 Kenya Medical Research Institute/Wellcome Trust Research Programme, Centre for Geographic Medicine Research – CoastKilifi, Kenya; 2 Health Protection AgencyLondon, UK; 3 Department of Biological Sciences, University of Warwick, CoventryUK

**Keywords:** respiratory syncytial virus, risk factors, disease, Kenya

## Abstract

**Objectives:**

To identify factors associated with developing severe respiratory syncytial virus (RSV) pneumonia and their commonality with all-cause lower respiratory tract infection (LRTI), in order to isolate those risk factors specifically associated with RSV-LRTI and identify targets for control.

**Methods:**

A birth cohort of rural Kenyan children was intensively monitored for acute respiratory infection (ARI) over three RSV epidemics. RSV was diagnosed by immunofluorescence of nasal washings collected at each ARI episode. Cox regression was used to determine the relative risk of disease for a range of co-factors.

**Results:**

A total of 469 children provided 937 years of follow-up, and experienced 857 all-cause LRTI, 362 RSV-ARI and 92 RSV-LRTI episodes. Factors associated with RSV-LRTI, but not RSV-ARI, were severe stunting (*z*-score ≤−2, RR 1.7 95%CI 1.1–2.8), crowding (increased number of children, RR 2.6, 1.0–6.5) and number of siblings under 6 years (RR 2.0, 1.2–3.4). Moderate and severe stunting (*z*-score ≤−1), crowding and a sibling aged over 5 years sleeping in the same room as the index child were associated with increased risk of all-cause LRTI, whereas higher educational level of the primary caretaker was associated with protection.

**Conclusion:**

We identify factors related to host nutritional status (stunting) and contact intensity (crowding, siblings) which are distinguishable in their association with RSV severe disease in infant and young child. These factors are broadly in common with those associated with all-cause LRTI. The results support targeted strategies for prevention.

## Introduction

Pneumonia is the leading cause of morbidity and mortality in the developing world (Bryce *et al.* 2005; WHO 2006a; Greenwood *et al.* 2007) and respiratory viruses make a major contribution to this disease burden (Selwyn 1990; Monto 2002). Among the viruses, RSV is a major contributor to community acquired pneumonia (Weber *et al.* 1998b, 2002; Robertson *et al.* 2004; Nokes *et al.* 2008). However, as with most viral infections, RSV is generally characterized by a self-limiting mild illness episode, and only a few patients will progress to severe or life-threatening conditions. The factors which influence this progression from infection to severe disease are not well understood.

Pneumonia has multiple aetiologies, and the risk factors for RSV-associated disease may be common to all pneumonias, specific to viral pneumonias or may be agent-specific. Past studies report a number of possible risk factors for increased viral disease severity, most of which are common to RSV. These may be environmental [higher household population density (Aaby 1988; Weber *et al.* 1999; Suwanjutha *et al.* 2002), attending school (Monto & Sook 1971; Hall *et al.* 1976), increased smoke exposure (Gardner *et al.* 1984; Cruz *et al.* 1990)], host factors [e.g. born prematurely (Nielsen *et al.* 2003), genetic susceptibility (Karron *et al.* 1999), poor nutritional status (Vardas *et al.* 1999; Loscertales *et al.* 2002; Djelantik *et al.* 2003)] and pathogen (Mufson *et al.* 1988; Sullender 2000) specific factors. Severe RSV in developing countries has been strongly associated with crowding (Weber *et al.* 1999), but this is not so for malnutrition, and in a number of instances malnutrition has been associated with protection (Adegbola *et al.* 1994; Nwankwo *et al.* 1994; Loscertales *et al.* 2002; Djelantik *et al.* 2003). Importantly all previous studies have the limitation of not distinguishing risk factors that are specific to disease as opposed to infection resulting in any level of severity.

We undertook a study to examine a range of potential risk factors for RSV disease (RSV-LRTI) in infants and young children followed from birth, within families under surveillance for ARI from a rural Kenyan community. Risk factors for progression from mild RSV infection to a LRTI were isolated by the extraction of factors identified for total RSV episodes irrespective of severity, and contrasted with factors associated with all-cause LRTI.

## Methods

The study was conducted in Kilifi, a rural district on the coast of Kenya with a tropical climate and seasonal rains (March–July and October–December). The community is served by a district hospital (KDH) based in Kilifi town. Ethical permission was provided by the Kenya National Ethical Review Committee and Coventry Research Ethics Committee, UK. The terminology used for respiratory disease throughout the text is described in Table 1.

**Table 1:** Terminology used for disease types

Term	Description and synonyms
LRTI	Lower respiratory tract infection. Used synonymously with pneumonia. Includes all cases identified as mild, severe or very severe pneumonia.
All-cause LRTI	Non-specific lower respiratory tract infection.
RSV-ARI	Clinical (i.e. symptomatic) RSV antigen positive episode. Irrespective of severity.
RSV-LRTI	RSV-associated LRTI (pneumonia) and equivalent to RSV disease.
Disease progression	Development of LRTI from upper respiratory tract infection.

### Birth cohort study

Full details of the birth cohort study have been described previously (Nokes *et al.* 2004, 2008; Okiro 2007). Briefly study participants were recruited between January 2002 and May 2003, from KDH maternity ward and the maternal child health clinic (if <2 weeks old), and if their homes were within easy access to the hospital. Written informed consent was obtained for participation. Surveillance continued until each child had experienced three RSV epidemics. These epidemics were clearly defined, occurring on an approximately annual basis, and lasting for between 14 and 21 weeks (mean 17 weeks) (Nokes *et al.* 2008). Households were visited by trained study field workers (FW) weekly during, and monthly outside of RSV epidemics. Potential cases of LRTI identified by a FW during home visits were referred to the clinic, and given one way bus fares. These referral cases were recognized by cough or difficulty in breathing (on the day or a history over the preceding week) in association with fast breathing for age (50 or more breaths per minute in infants, and 40 or more breaths per minute otherwise), or (alone or accompanied by) the presence of lower chest wall indrawing (World Health Organization 2005) or difficulty in breathing alone if observed on the day of the visit. ARI surveillance through presentation at the research out-patient (OP) clinic at KDH was maintained throughout the follow-up period either by self (passive) referral, or FW (active) referral at home visits. To enhance passive surveillance (self referral), mothers were encouraged to bring their child to the research clinic if they identified any symptoms of respiratory infection. At the OP clinic, the severity of respiratory disease was ascribed following a review by a study clinician, which would include (a repeat) measurement of respiratory rate. Transport costs were reimbursed, and definitive medicines were provided without charge. At each contact with study participants identification of symptoms consistent with ARI on the day or during the preceding week, and an absence of RSV infection for the prior 14 days, prompted the collection of a nasal specimen by nasal washing. Specimens were examined for RSV antigen by direct immunofluorescence test (DFA, Chemicon). The severity of respiratory disease was ascribed following a clinical review using a standard proforma and based on WHO guidelines (Nokes *et al.* 2004; WHO 2005).

### Risk factor survey

Between June and November 2004 a cross-sectional risk factor survey was carried out on households of all birth-cohort children remaining under surveillance. The purpose of the study was explained to the parents or guardians and verbal consent sought before the interview commenced. A household was defined as all individuals who normally eat at the same meal. Individuals 15 years of age or older were considered adults. The questionnaire was based on previous risk-factor surveys conducted in sub-Saharan Africa (Ballard & Neumann 1995; Weber *et al.* 1999; Broor *et al.* 2001) addressing household characteristics, and demographic, socioeconomic and environmental factors, but tailored to the specific setting of the study community. Data related to key asset indicators including primary caretaker (PCT) education level, occupation of the major income provider (MIP), housing characteristics (type of walls, sanitation), source of drinking water, family size and sleeping patterns in relation to the birth cohort child and nutritional status was collected. Crowding and contact intensity was measured by the total household size, number of sibling children in the household, and sleeping proximity.

A wealth index was constructed from data on household asset ownership (e.g. owning a bicycle) and characteristics (e.g. house and toilet type) using principal component analysis (Filmer & Pritchett 2001). Weights (scoring coefficients) derived from the first principal component were used to assign each household a wealth index from which socioeconomic groups were defined as follows: the top 33% were referred to as ‘least poor’, the next 33% as ‘poor’ and the bottom 34% as ‘most poor’.

Anthropometric measurements were obtained at birth and at 3-month intervals thereafter for cohort children. A WHO macro [igrowup_STATA macro (WHO 2006b) ] was used to calculate *z*-scores (the standardized deviation from the median of a reference population) for three anthropometric indicators: weight-for-age (waz-underweight), length or height-for-age (haz-stunting), weight-for-length or height (whz-wasting).

### Data analysis

Data were double entered onto FileMaker (FileMaker Pro 5.5 v1) with internal consistency checks, and analysed using Stata (v8.2, STATACorp, Texas). Longitudinal data on infection history were combined with cross-sectional data from the risk factor survey. Observation time included days from date of recruitment until the last study visit, or until lost to follow-up, excluding days absent from the district. Each child had multiple record visit data over the follow-up period. Certain variables were reassigned at intervals of 3 months (nutritional status) or at each epidemic period (number of siblings sleeping in house, rooms and beds).

For the purpose of analysis only clinical data obtained from CO reviews were used (as opposed to that of the FW) and a diagnosis of LRTI was assigned to children with acute cough or difficulty in breathing in association with any one or more of the following (i) raised respiratory rate for age (respiratory rate of ≥40 breaths/min for children aged >12 months, ≥50 breaths/min for ages greater than 1 month, and ≥60 for a child of any age), (ii) lower chest wall indrawing or (iii) inability to feed, reduced conscious level or hypoxia (O_2_ saturation <90% by Oximetry), the latter group only if confirmed by the clinician’s own diagnosis of LRTI or bronchiolitis. The outcome variables were: (i) all-cause LRTI, (ii) RSV-ARI and (iii) RSV-LRTI (as defined in Table 1).

Univariate analysis was performed to describe the study population and identify risk factors for inclusion in multivariate analysis. Predictors were considered for inclusion in the multiple regression models using the log-rank test of equality of survival distribution across strata (for categorical variables) or a univariate Cox proportional hazard regression for the continuous variables. Predictors were considered for inclusion if the test had a *P*-value of 0.25 or less, and for groups of collinear variables (e.g. household contact measures) only those with the strongest univariate association were included. Significant variables were included in the multivariate models using a non-automated forward stepwise regression starting from the variable with the highest test statistic. Variables that no longer showed significance (*P* ≥ 0.05) were removed. For highly correlated variables (*r* ≥ 0.8) only the variable remaining significant in the multivariate model was included. The Cox shared frailty model was used with the all-cause LRTI outcome because of significant multiple failures per individual (*θ* = 0.326, *P* < 0.001). The standard Cox model with adjusted standard errors adjusting for clustering within individual was used for RSV-ARI and RSV-LRTI. Analysis time was calendar time, eliminating the potential confounding effect of seasonality in RSV and all-cause LRTI. Time-varying covariate(s) were specified through multiple observations per subject, ensuring risk sets at each failure were associated with the correct value of the risk factor. The results are reported as relative risks (hazard ratios) with 95% confidence intervals.

## Results

**Figure 1:**
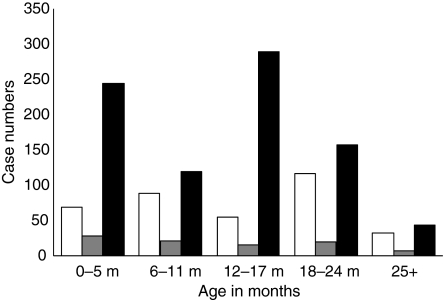
Descriptive characteristics of cases identified during the study. The bars represent the absolute numbers of cases of RSV-ARI (white bars), RSV-LRTI (grey bars) and all-cause LRTI (black bars) by specific age category.

**Figure 2:**
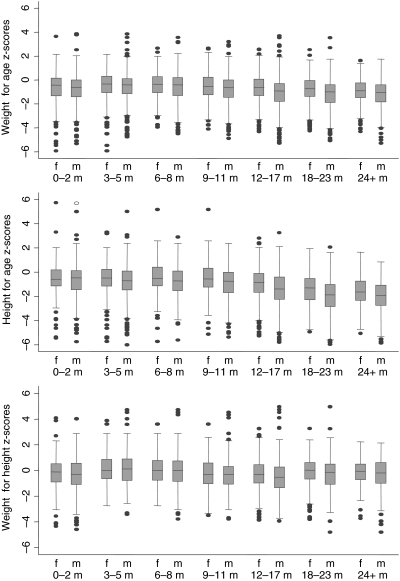
Weight for age, height for age, weight for height *z*–scores of children in the study by sex and age. The *box plot* depicts the interquartile range as a *box* and the median as a line in the box. *Bars*, upper and lower adjacent values and dots represent outliers.

The birth cohort was monitored over four calendar years until each child had lived through three epidemics of RSV infection. From the 469 children under surveillance at the time of the risk factor survey, 29 979 separate visits (observations) for the detection of all-cause LRTI, RSV-ARI and RSV-LRTI were made. The observations per child were between 23 and 104 with a mean and median of 71. There were 362 episodes of clinical RSV-ARI detected among 940 person-years of observation; 283 single infections and 79 children were identified as being symptomatically infected by RSV more than once (68 twice, 8 three times, 2 four times and 1 five times). There were 857 episodes of all-cause LRTI; 128 (37%) children had only one episode of LRTI, 216 (63%) had two or more episodes. Of the all-cause LRTI episodes, 92 were associated with RSV; 86 (93%) children had a single episode of RSV-LRTI, with 6 children having two episodes. Population characteristics for the cohort are detailed in Table 2. There were more cases of RSV infection and RSV-LRTI in females than males; 55% and 52%, respectively, while cases of all-cause LRTI were equally distributed by sex. The age distribution of cases for all the three outcomes is shown in Figure 1. The mean age of a child with a RSV infection was 13.9 months (median age, 13 months) while that for a RSV-associated LRTI was 11.7 months (median age, 10 months). The mean age of a child with all-cause LRTI was 11.8 months (median age, 12 months). Figure 2 illustrates the different nutritional indices summarized by age progressively through follow-up.

**Table 2:** Model parameters and selected characteristics of the study population

			Number of events for each outcome		
			
Parameters	Percent of population†	Pyo	RSV ARI	RSV-LRTI	All-cause LRTI
Current age (months)‡					
0–5	21.0¶	200.5	69	28	245
6–11	22.4	213.2	88	21	120
12–17	22.1	211.3	55	16	290
18 or more	34.5	315.2	150	27	202
Multiple babies					
1 child	93.8	880.5	337	83	790
Twins	5.8	55.1	22	8	50
Triplets	0.4	4.6	3	1	17
Educational level of PCT (years)					
No schooling	25.4	241.0	92	25	249
1–7	39.2	370.2	156	31	353
8–12	31.1	289.5	105	34	241
>12	4.3	3839.5	9	2	14
PCT age group (years)					
13–20	14.9	136.5	49	26	125
21–30	48.8	458.7	186	24	428
31–40	27.7	262.4	92	28	226
41–50	6.6	63.0	23	14§	51
51–63	1.9	19.6	12		27
Child’s care					
Mother	81.2	762.2	300	73	670
Other family members	12.2	114.3	41	13	129
House help	4.3	39.5	7	3	20
School	0.2	2.4	1	0	0
Mother/family member	2.1	21.7	13	3	38
Smokers in HH					
None	71.9	673.1	255	66	597
1	23.5	223.8	83	21	216
2 or more	4.7	43.3	24	5	44
Family assisted††					
No	19.4	756.7	273	69	665
Yes	80.6	183.5	89	23	192
House and toilet type					
Mudwall no toilet	29.9	288.0	124	26	287
Blockwall no toilet	3.2	31.1	15	7	31
Mudwall latrine	30.9	290.4	121	34	303
Blockwall latrine	29.2	268.8	88	24	211
Mudwall flush toilet	0.6	4.7	1	0	0
Block flush toilet	6.2	57.2	13	1	25
Main cooking fuel					
Gas/paraffin	3.8	35.9	11	0	16
Charcoal	23.5	211.4	66	12	158
Firewood	70.2	669.6	276	79	660
Firewood/charcoal	2.6	23.3	9	1	23
Job category description of MIP					
Non-skilled	37.3	354.2	145	35	359
Trade	16.6	156.6	74	21	146
Skilled	35.4	330.0	115	27	284
Professional	10.7	99.4	28	9	68
Weight for age z-score‡					
>−1	61.6¶	579.8	214	49	507
−1.99 to −1	24.3	228.2	92	27	205
≤−2	14.0	132.1	56	16	145
Height for age z-score‡					
>−1	49.3¶	458.3	162	38	363
−1.99 to −1	26.1	247.0	97	26	239
≤−2	24.7	234.9	103	28	255
Weight for height z-score‡					
>−1	76.9¶	725.0	280	71	652
−1.99 to −1	17.2	160.3	56	13	136
≤−2	5.9	54.9	26	8	69
Family children					
1–5	72.7	681.4	257	60	598
6–10	24.1	231.2	92	26	214
11 or more	3.2	27.6	13	6	45
Siblings <6 years					
None	28.6	262.8	88	16	185
1–2	56.5	536.9	212	58	517
3–4	12.6	119.5	53	15	129
5 or more	2.4	21.0	9	3	26
Number of siblings <6 years sleeping in same room as index‡					
None	55.8¶	524.3	190	50	425
1–2	44.1	404.8	164	39	421
3	1.2	11.0	8	3	11
Number of siblings 6 years or over sleeping in same room as index‡					
None	67.0¶	629.8	250	63	551
1	19.4	183.8	70	19	203
2–3	11.7	108.4	34	8	90
4	1.9	18	8	2	13
Number of siblings <6 years going to school‡					
None	83.4¶	780.7	289	77	732
1	13.8	130.8	53	10	87
2–3	2.8	28.7	20	5	38

Index refers to the birth cohort child.

Pyo, person years of observation; PCT, primary caretaker; MIP, major income provider; HH, household.

† Proportion of the study population with this characteristic. Excludes factors that were reassigned during study period.

‡ Time changing variables.

§ PCT aged 41 to 63 years.

¶ Proportion of total observation time spent in specific category.

†† Family assisted means the family receives financial assistance from relatives living away from home.

Factors significantly associated with the risk of acquiring RSV-ARI and RSV-LRTI or all-cause LRTI by univariate analysis are presented in Table 3. Increased risk arose with higher number of children in the household and number of siblings under 6 years of age as well as more male siblings. Having one or more siblings sleeping in the same room as the birth cohort child was associated with increased risk of all-cause LRTI while having one or more siblings <6 years sleeping in the same bed as the birth cohort child was associated with increased risk of RSV-ARI. Moderate-to-severe malnutrition was associated with increased risk of RSV-LRTI (haz ≤ −2) and LRTI (waz and whz ≤ −2 and haz ≤ −1). Having two or more smokers in the household was correlated with increased risk of infection. Living in a mud-walled house, using firewood as the main cooking fuel and being a child of a multiple birth was associated with increased risk of RSV-ARI and all-cause LRTI. Having an older PCT was also associated with increased risk of infection and RSV-LRTI. Several factors were related to protection: living in a household classified as ‘least poor’, having a hired house help, a flush toilet, a major income provider with a professional job or a PCT with higher than high school level education.

**Table 3:** Univariate analysis of risk factors associated with infection and disease

		Relative risk (95% CI)		
		
Putative risk factor	Categories	RSV-ARI	RSV-LRTI	All-cause LRTI
Socioeconomic status	Most poor	–	–	–
	Poor	1.02 (0.83–1.26)	1.49 (0.94–2.37)	1.02 (0.81–1.28)
	**Least poor**	**0.75 (0.60–0.95)**	0.80 (0.48–1.34)	**0.70 (0.55–0.89)**
Current age (months)	0–5	–	–	–
	**6–11**	1.44 (0.74–2.80)	1.20 (0.38–3.77)	**0.58 (0.43–0.77)**
	**12**–**17**	1.16 (0.54–2.45)	1.44 (0.38–5.39)	**1.85 (1.40–2.43)**
	18 or more	0.98 (0.48–2.00)	1.06 (0.28–4.05)	0.85 (0.59–1.21)
Multiple delivery	1 child	–	–	–
	Twins	1.05 (0.73–1.51)	1.54 (0.79–3.01)	1.02 (0.75–1.34)
	**Triplets**	**1.77 (1.06–2.93)**	1.99 (0.47–8.50)	**3.85 (3.35–4.42)**
Age group of PCT (years)	13–22	–	–	–
	23–30	1.14 (0.85–1.51)	2.15 (0.84–5.47)	0.99 (0.74–1.31)
	31–40	0.98 (0.71–1.35)	2.43 (0.94–6.27)	0.93 (0.68–1.28)
	**41**–**50**	0.96 (0.62–1.48)	**3.65 (1.34–9.98)**†	0.88 (0.58–1.32)
	**51–63**	**1.73 (1.16–2.59)**		1.36 (0.96–1.92)
Education level of PCT (years)	No schooling	–	–	–
	1–7	1.11 (0.88–1.39)	0.84 (0.49–1.44)	0.94 (0.80–1.11)
	8–12	0.96 (0.76–1.21)	1.14 (0.68–1.92)	0.80 (0.63–1.03)
	**>12**	0.61 (0.36–1.04)	0.51 (0.13–2.04)	**0.34 (0.21–0.55)**
Literate PCT	No	–	–	–
	**Yes**	1.00 (0.83–1.21)	0.96 (0.63–1.46)	**0.82 (0.67–0.98)**
Family assisted financially	No	–	–	–
	**Yes**	**1.34 (1.11–1.63)**	1.40 (0.91–2.15)	1.20 (0.97–1.49)
Weight-age-*z* score	>−1	–	–	–
	−1.99 to −1	0.99 (0.80–1.24)	1.37 (0.86–2.17)	1.06 (0.90–1.25)
	**≤**−**2**	1.01 (0.79–1.29)	1.37 (0.82–2.28)	**1.30 (1.08–1.56)**
Height-age-*z* score	>−1	–	–	–
	−**1.99 to**−**1**	1.07 (0.85–1.36)	1.43 (0.87–2.36)	**1.32 (1.12–1.56)**
	≤−**2**	1.18 (0.95–1.47)	**1.85 (1.15–2.97)**	**1.60 (1.36–1.89)**
Weight-height-*z* score	>−1	–	–	–
	−1.99 to −1	0.82 (0.63–1.07)	0.70 (0.40–1.26)	0.91 (0.76–1.10)
	≤−**2**	0.97 (0.69–1.36)	1.17 (0.58–2.37)	**1.41 (1.10–1.81)**
Number of family children	1–5	–	–	–
	6–10	1.04 (0.84–1.27)	1.32 (0.85–2.04)	1.08 (0.86–1.37)
	**11 or more**	1.09 (0.87–1.36)	**2.51 (1.32–4.76)**	**1.80 (1.31–2.47)**
Number siblings under 6 years	None	–	–	–
	**1–2**	1.17 (0.93**–**1.47)	**1.78 (1.06–2.98)**	**1.37 (1.16–1.62)**
	**3–4**	**1.32 (1.00–1.73)**	**2.00 (1.00–3.97)**	**1.50 (1.21–1.88)**
	**5 or more**	1.34 (0.87–2.06)	2.39 (0.81–7.09)	**1.70 (1.28–2.26)**
Number of male siblings	0–2	–	–	–
	3–6	1.09 (0.88–1.34)	1.36 (0.87–2.10)	1.22 (0.97–1.54)
	**7 or more**	1.36 (0.98–1.88)	**3.28 (1.76–6.17)**	1.15 (0.65–2.04)
Index child’s care	Mother	–	–	–
	Another family member	0.91 (0.68–1.21)	1.12 (0.63–1.98)	1.24 (0.99–1.56)
	**House help**	**0.45 (0.24–0.81)**	0.78 (0.27–2.31)	**0.56 (0.36–0.86)**
	School	**1.13 (1.01–1.26)**	–	–
	**Mother/family member**	1.52 (0.95–2.43)	1.28 (0.48–3.38)	**1.89 (1.22–2.93)**
House type	Block walled	–	–	–
	**Mud walled**	**1.29 (1.07–1.56)**	1.15 (0.76–1.76)	**1.37 (1.12–1.67)**
House ownership	Owner occupied	–	–	–
	**Rented**	0.83 (0.64–1.07)	**0.52 (0.28–0.96)**	0.87 (0.67–1.14)
	Not owned/ rented	0.94 (0.62–1.42)	0.55 (0.15–2.00)	0.62 (0.35–1.10)
Toilet type	No toilet	–	–	–
	**Flush**	**0.52 (0.34–0.78)**	0.16 (0.02–1.13)	**0.40 (0.24–0.67)**
	Latrine	0.85 (0.71,1.03)	1.03 (0.68,1.57)	0.93 (0.76,1.14)
Main fuel used for cooking	Charcoal	–	–	–
	Gas/paraffin	1.07 (0.61–1.88)	–	1.61 (0.96–2.68)
	**Firewood**	1.37 (0.81–2.33)	**2.08 (1.18–3.66)**	**2.16 (1.35–3.46)**
	Firewood/charcoal	1.26 (0.53–2.97)	0.82 (0.13–5.29)	**2.27 (1.10–4.69)**
Water source site	Public	–	–	–
	**Own source**	1.19 (0.85–1.66)	**0.22 (0.06–0.86)**	**2.12 (1.50–3.00)**
	Shared	1.17 (0.79–1.72)	0.71 (0.38–1.31)	**2.07 (1.37–3.11)**
job description of MIP	Non-skilled	–	–	–
	Trade	1.17 (0.93–1.48)	1.42 (0.84–2.40)	0.94 (0.71–1.24)
	Skilled	0.85 (0.68–1.06)	0.84 (0.53–1.34)	0.86 (0.68–1.07)
	**Professional**	**0.71 (0.52–0.96)**	0.97 (0.46–2.03)	**0.69 (0.53–0.91)**
Number of smokers in Household	None	–	–	–
	1	0.99 (0.78–1.24)	0.93 (0.56–1.53)	1.08 (0.87–1.35)
	**2 or more**	**1.47 (1.13–1.93)**	1.21 (0.47–3.11)	1.15 (0.81–1.64)
Number of adults sleeping in index’s room	0–1	–	–	–
	**2**	1.02 (0.84–1.25)	**0.66 (0.44–1.00)**	0.82 (0.66–1.02)
	3 or more	0.87 (0.55–1.39)	0.82 (0.34–1.95)	0.88 (0.52–1.49)
Number of siblings <6 years living in same house as index	None	–	–	–
	1	1.07 (0.87–1.30)	1.00 (0.65–1.55)	1.10 (0.89–1.35)
	**2**–**4**	1.01 (0.78–1.31)	**1.73 (1.02–2.93)**	**1.41 (1.06–1.86)**
Number of siblings 6 years or over living in same house as index	None	–	–	–
	1–2	1.02 (0.84–1.25)	0.92 (0.59–1.44)	1.14 (0.92–1.42)
	3–4	0.87 (0.67–1.12)	0.91 (0.49–1.69)	0.86 (0.66–1.12)
	**5**–**7**	0.98 (0.65–1.43)	**2.10 (1.08–4.10)**	1.45 (0.90–2.36)
Number of siblings <6 years sleeping in same room as index	None	–	–	–
	**1–3**	1.08 (0.91–1.30)	1.05 (0.71–1.55)	**1.30 (1.07–1.57)**
Number of siblings 6 years or over sleeping in same room as index	None	–	–	–
	1	0.96 (0.74–1.25)	1.05 (0.64–1.71)	**1.27 (1.00–1.62)**
	2–3‡	0.81 (0.62–1.05)	0.77 (0.39–1.53)	0.95 (0.72–1.26)
	4			0.83 (0.38–1.29)
Number of siblings <6 years sleeping in same bed as index	None	–	–	–
	**1–3**	1.16 (0.78–1.72)	1.22 (0.99–1.50)	**1.21 (1.01–1.45)**
Number of siblings <6 years going to school	None	–	–	–
	**1**	0.88 (0.68–1.12)	0.75 (0.40–1.41)	**0.77 (0.60–0.98)**
	**2–3**	1.40 (0.99–2.00)	1.77 (0.76–4.12)	**1.69 (1.02–2.80)**
Number of siblings 6 years or over going to school	None	–	–	–
	1–2	0.97 (0.78–1.19)	1.02 (0.61–1.70)	1.13 (0.87–1.44)
	3–5	0.97 (0.76–1.25)	1.31 (0.78–2.20)	1.12 (0.84–1.48)
	**6**–**9**	1.17 (0.76–1.80)	**3.04 (1.48–6.22)**	1.46 (0.98–2.26)

Significant estimates (*P* < 0.05) are in bold.

MIP, major income provider; PCT, primary caretaker.

† PCT aged 41 to 63 years.

‡ 2–4 siblings 6 years or over sleeping in same bed as index for RSV ARI and RSV LRTI.

Results from the multivariate regression are shown in Table 4. Factors independently associated with RSV-ARI are shown in column 1. Higher age of the PCT (>50) was the strongest independent predictor of increased risk. Exposure to tobacco smoke was also associated with an increased risk, whereas two indicators of higher socioeconomic status (SES), namely, block-walled house with a flush toilet and hired house help with child care, were associated with protection from RSV-ARI.

**Table 4:** Risk factors independently predicting increased relative risks of infection and disease

		Relative risk (95% CI)		
Risk factor	Categories	RSV-ARI	RSV-LRTI	All cause -LRTI
Current age (month)	0–5	–	–	1
	**6–11**	–	–	**0.55 (0.41–0.74)**
	**12–17**	–	–	**1.72 (1.31–2.25)**
	18 or more	–	–	0.74 (0.52–1.04)
Multiple babies	1 child	–	–	1
	Twins	–	–	0.80 (0.54–1.19)
	**Triplets**	–	–	**4.12 (1.55–10.9)**
Education level of PCT(years)	No schooling	–	–	1
	1–7	–	–	0.95 (0.76–1.19)
	8–12	–	–	0.81 (0.64–1.19)
	**>12**	–	–	**0.40 (0.21–0.76)**
Child’s care	Mother	1	1	–
	Another family member	0.77 (0.54–1.08)	0.95 (0.53–1.71)	–
	**House help**	**0.54 (0.30–0.99)**	1.49 (0.56–4.00)	–
	School	1.06 (0.87–1.30)	0.00 (–)	–
	Mother / family member	1.28 (0.80–2.05)	1.26 (0.47–3.37)	–
PCT age group (years)	13–24	1	1	–
	25–30	1.15 (0.87–1.52)	1.03 (0.58–1.82)	–
	31–40	1.09 (0.79–1.49)	1.49 (0.88–2.53)	–
	41–50	1.02 (0.66–1.57)	**2.19 (1.13–4.25)**†	–
	**51–63**	**2.13 (1.27–3.59)**		–
Number of smokers in HH	None	1	1	–
	1	0.92 (0.74–1.15)	0.87 (0.53–1.45)	–
	**2 or more**	**1.40 (1.07–1.84)**	0.81 (0.36–1.84)	–
Height-for-age *z*-score	>−1	–	1	1
	−**1.99 to**−**1**	–	1.34 (0.83–2.17)	**1.27 (1.06–1.52)**
	**≤**−**2**	–	**1.73 (1.08–2.76)**	**1.38 (1.12–1.70)**
Family assisted	No	1	1	–
	Yes	1.20 (0.98–1.46)	1.34 (0.86–2.09)	–
House and toilet type	Mud wall no toilet	1	1	–
	**Block wall no toilet**	1.03 (0.73–1.46)	**3.62 (1.53–8.88)**	–
	Mud wall with latrine	0.98 (0.79–1.21)	1.57 (0.96–2.57)	–
	Block wall with latrine	0.83 (0.65–1.06)	1.41 (0.79–2.53)	–
	Mud wall flush toilet	0.53 (0.19–1.47)	0.00 (−)	–
	**Block flush toilet**	**0.60 (0.40–0.91)**	0.25 (0.03–2.11)	–
Main cooking fuel	Gas/paraffin	–	–	1
	Charcoal	–	–	1.34 (0.74–2.44)
	Firewood	–	–	1.71 (0.96–3.05
	Firewood/charcoal	–	–	2.11 (0.97–4.60)
Job description of MIP	Non-skilled	–	1	–
	Trade	–	1.64 (0.98–2.73)	–
	Skilled	–	0.91 (0.56–2.09)	–
	Professional	–	1.04 (0.48–2.27)	–
Number of family children	1–5	–	1	1
	6–10	–	0.97 (0.57–1.66)	0.96 (0.77–1.19)
	**11 or more**	–	**2.58 (1.03–6.50)**	**1.68 (1.07–2.63)**
Number of siblings under 6 years	None	–	1	–
	**1**–**2**	–	**2.00 (1.17–3.42)**	–
	3–4	–	1.99 (0.81–4.91)	–
	5 or more	–	1.74 (0.54–5.63)	–
Number of siblings <6 years sleeping in same room as index	None	–	–	1
	1 –2	–	–	1.19 (0.99–1.43)
	3	–	–	1.69 (0.81–3.51)
Number of siblings 6 years or more sleeping in same room as index	None	–	–	1
	**1**	–	–	**1.29 (1.04–1.61)**
	2–3	–	–	0.83 (0.62–1.11)
	4	–	–	0.63 (0.31–1.28)
Number of siblings <6 years going to school	None	–	–	1
	1	–	–	0.80 (0.62–1.05)
	2–3	–	–	1.52 (0.98–2.33)

Statistically significant results (*P* < 0.05) are in bold.

MIP, major income provider; PCT, primary caretaker.

† PCT aged 41 to 63 years.

Significant predictors of the risk of infection were included in the RSV-LRTI multivariate analysis as the baseline. Additional variables linked to RSV-LRTI from the univariate analysis were then fitted, and independent predictors reported in column 2 of Table 4. Of the predictors identified to increase the risk of clinical RSV-ARI, increased age of PCT (>40 years) and the house and toilet type (block wall with no toilet) were also associated with an increase in risk of RSV disease. Crowding (as measured by number of children in the home) and number of children under 6 years in the home were found to correlate with increased risk of RSV-LRTI. Moderate-to-severe stunting (height-for-age *z*-score ≤ −2) was also an independent predictor of RSV-LRTI.

To identify which risk factors for disease were specific to RSV, we determined predictors of all-cause LRTI in this study population (Table 4, column 3). Factors found to be independently associated with increased risk of all-cause LRTI were height-for-age *z*-score of ≤ −1, crowding (number of children in the home), and contact pattern (number of siblings over 6 years of age sleeping the same room as cohort child). Current age was also an independent predictor of all-cause LRTI associated with protection in those 6–11 months and increased risk in those 12–17 months. A multiple birth (triplets) was the strongest independent predictor of LRTI, although rare in the cohort (Table 2). A borderline significant factor was having 2–3 siblings <6 years attending school. A caretaker with a college education (>12 years of schooling) was associated with reduced risk of all-cause LRTI.

## Discussion

Data collected over 4 years of follow-up of a large birth cohort in rural Kenya were analysed to determine risk factors specific for RSV-ARI and those specific to RSV disease, and these were compared with factors common to all-cause LRTI. Two main factors emerged as being independently associated with increased risk of severe disease (both RSV-LRTI and all-cause LRTI) as opposed to total ARI, namely, growth stunting and household crowding.

The data show that stunting (mild to moderate and severe), an indication of long-term malnutrition, was a more important factor for RSV-LTRI and all-cause LRTI than acute (short-term) malnutrition (wasting). This risk has previously been reported in a study involving Kenyan children (Ballard & Neumann 1995). It is thought that malnourished children may be susceptible to opportunistic infections; although concurrent RSV and bacterial infections are uncommon (Weber *et al.* 1998a; Madhi *et al.* 2001; Loscertales *et al.* 2002; Nokes *et al.* 2008). Results from several studies indicate a deficiency in the immune response in malnourished children (Neumann *et al.* 1975; Chandra 1983; Watson *et al.* 1985). One other study in South Africa (Vardas *et al.* 1999) also reported increased risk of RSV-LRTI in admissions with malnutrition. However, there are several reports of an absence of association between, or a protective effect of, malnutrition and the incidence of RSV disease (Adegbola *et al.* 1994; Nwankwo *et al.* 1994; Miranda-Novales *et al.* 1999; Loscertales *et al.* 2002; Djelantik *et al.* 2003). Conversely, malnutrition is a widely known risk factor for ARI and all-cause LRTI (Selwyn 1990; Tupasi *et al.* 1990; Ballard & Neumann 1995). These differences between studies are hard to reconcile because of different methodologies, notably whether hospital-based or community-based and differences in definition used for malnutrition and adjustments for covariates. Notwithstanding these differences, our database represents the largest ever RSV community study, and together with a strong analysis design and properly computed *z*-scores (WHO 2006b), give the results credibility. The influence of HIV as a confounder of this association is not precisely known. Overall HIV-1 prevalence in women attending to KDH antenatal care in 2004 was 4.8% (95% CI 3.4–6.6) (E. Sanders, personal communication).

The second important predictor of disease was crowding, as measured by number of children, and siblings under 6 years of age, which was associated with increased risk of RSV-LRTI and all-cause LRTI. The underlying mechanism may be increased contact intensity, resulting in an increase in size and/or duration of the exposure inoculum resulting from proximity of contact (intimacy of contact) (DeVincenzo 2005). The association between proximity of contact and disease severity has been well described for measles in Guinea Bissau (Aaby *et al.* 1983; Aaby & Coovadia 1985; Aaby 1988). Severity of measles infections increases when two or more children are sick simultaneously, and where the secondary infected child sleeps in close proximity to the child who introduced the infection into the household. Likewise several recent studies (Simoes 2003) found a positive association between crowding and number of siblings and occurrence of RSV-LRTI. An alternative mechanism could be related to contact frequency such that children in contact with more people are exposed to more inocula (sequential inoculation from numerous contacts), which increases their chance of severe disease.

Several factors related to intensity (family size) and pattern of contact (sleeping arrangements; in same house, room, bed or with school going sibling) between other children in the home and the cohort child were investigated. An association between the number of siblings sleeping in the same room as the cohort child and increased risk of all-cause LRTI was observed (Table 4). Several studies have shown a similar correlation between risk of RSV-LRTI and all-cause LRTI and number of people sleeping in the same room with the child or with siblings in school (Aaby *et al.* 1984; Holberg *et al.* 1991; Suwanjutha *et al.* 2002; Cardoso *et al.* 2004). This reflects the higher probability of transmission taking place due to prolonged exposure and closer contact; thought necessary for RSV transmission (Hall & Douglas 1981; Aaby & Coovadia 1985). Similarly, RSV infection and illness rates were found to be higher in mothers than fathers as presumably mothers had more intimate contact with the children than did the fathers (Berglund 1967) – again suggesting that virus dosage and exposure time may play an important part in the outcome of RSV infection.

Other factors significantly associated with individual outcomes in the multivariate analysis included house and toilet type which relates to SES, exposure to tobacco and environmental smoke from cooking fuel (borderline significance) which have previously been reported (Pandey *et al.* 1989; Cruz *et al.* 1990) as increasing the risk of RSV-ARI and LRTI exposure to cooking smoke is prevalent due to mothers carrying their young children while attending to household chores (field observations). Interestingly, the risk of all-cause LRTI decreased with increased level of education of the PCT. Unfortunately, less than 5% of the mothers included in this study had more than a high school education with 25% having had no schooling at all.

Clustering of outcomes was observed in these data with several children experiencing more than one episode of LRTI or RSV-specific LRTI pointing to the role of some host-specific factors. This clustering was significant for all-cause pneumonia with 63% of the study population experiencing two or more episodes of LRTI.

The magnitude of a risk at the population level is related to its prevalence. For instance, although children in households with 11 or more occupants are at increased risk of severe RSV disease (RR 2.6), only 3.3% of households have this many occupants. In contrast, having 1–2 siblings under 6 years is of lower risk (RR 2) but 56.5% of the population fall into this category.

A possible limitation of this study is that diagnosis of RSV infection on the basis of antigen detection alone has lower sensitivity compared with the use of assay combinations (Hall *et al.* 1976; Glezen *et al.* 1986). However, the sensitivity of the immunofluorescence antigen test is related to the concentration of antigen in the sample, so that infections undiagnosed are more likely to be milder. Consequently, the risk factors we have found are strictly associated with RSV infection detectable by our methods. The strength of the study was the re-specification of time-varying covariates during the study period reducing possible misclassification of exposure.

In conclusion, our results strongly suggest poor host nutritional status (severe stunting) and household size (high contact intensity and sibling numbers) as significant risk factors for severe RSV disease, and that these are broadly common to all-cause LRTI. These data not only reinforce previously suspected associations but also, through careful study design, provide more specific evidence for the relationship with disease progression. These data have implications for our understanding of RSV transmission and control. The notion of differences in the risk of acquiring disease according to contact age and intensity suggests the importance of these factors in transmission and control particularly in relation to who acquires infection from whom. In the light of this, vaccination programmes targeted to school children, who constitute the siblings within households, may show promise by providing indirect protection to the infant. Additional benefits would be achieved from education of mothers on reducing the intensity of contacts between siblings and the naïve young infant who is at most risk of severe disease. Furthermore, an increased risk of pneumonia in those with chronic poor nutritional status reinforces the need for community-based interventions directed towards improved diet, supplementation (vitamin supplements or fortified milk) and parental education (promoting breastfeeding), already acknowledged to have significant positive benefits on all outcomes.
